# Role of Redox Homeostasis in the Communication Between Brain and Liver Through Extracellular Vesicles

**DOI:** 10.3390/antiox13121493

**Published:** 2024-12-06

**Authors:** Javier Huete-Acevedo, Cristina Mas-Bargues, Marta Arnal-Forné, Sandra Atencia-Rabadán, Jorge Sanz-Ros, Consuelo Borrás

**Affiliations:** 1 MiniAging Research Group, Department of Physiology, Faculty of Medicine, University of Valencia, CIBERFES, INCLIVA, Avenida Blasco Ibáñez, 15, 46010 Valencia, Spain; javier.huete@uv.es (J.H.-A.); cristina.mas@uv.es (C.M.-B.); mararfor@alumni.uv.es (M.A.-F.); sandra.atencia@uv.es (S.A.-R.); 2 Department of Pathology, Stanford University School of Medicine, Stanford, CA 94305, USA; jsanzros@stanford.edu

**Keywords:** extracellular vesicles, redox homeostasis, brain-liver axis

## Abstract

Extracellular vesicles (EVs) are small, membrane-bound particles secreted by cells into the extracellular environment, playing an increasingly recognized role in inter-organ communication and the regulation of various physiological processes. Regarding the redox homeostasis context, EVs play a pivotal role in propagating and mitigating oxidative stress signals across different organs. Cells under oxidative stress release EVs containing signaling molecules that can influence the redox status of distant cells and tissues. EVs are starting to be recognized as contributors to brain-liver communication. Therefore, in this review, we show how redox imbalance can affect the release of EVs in the brain and liver. We propose EVs as mediators of redox homeostasis in the brain-liver axis.

## 1. Introduction

### 1.1. Overview of Redox Homeostasis

Redox homeostasis refers to the delicate balance between oxidation and reduction (redox) processes in biological systems, ensuring cellular activities’ proper functioning and overall health. In living organisms, redox reactions are fundamental to various methods, including energy production, detoxification, and signal transduction [1]. Redox homeostasis lies in regulating reactive oxygen species (ROS) and reactive nitrogen species (RNS), which are byproducts of normal cellular metabolism. ROS and RNS include free radicals like superoxide anions, hydroxyl radicals, and non-radical molecules such as hydrogen peroxide (H_2_O_2_) and nitric oxide (NO) [2]. While these species are essential for several physiological functions, such as immune defense and cellular signaling, their overproduction can lead to oxidative stress, damaging lipids, proteins, and DNA [3,4].

Cells maintain redox homeostasis through a complex network of antioxidant defenses, which include enzymatic and non-enzymatic components. Key enzymatic antioxidants include superoxide dismutase (SOD), catalase, and glutathione peroxidase. These enzymes work to neutralize excessive amounts of ROS. Non-enzymatic antioxidants, such as vitamins C and E, glutathione, and flavonoids, are crucial in scavenging free radicals and preventing oxidative damage [5].

The balance between ROS production and antioxidant defenses is influenced by various factors, including environmental stressors (e.g., pollution and UV radiation), lifestyle choices (e.g., diet and smoking), and pathological conditions (e.g., inflammation and cancer) [6]. When this balance is disrupted, and the production of ROS and RNS overwhelms antioxidant defenses, oxidative and nitrosative stress ensues. Conversely, reductive stress occurs when there is an excess of reducing equivalents, such as NADH or glutathione, leading to an abnormal reduction in cellular components [7]. This can impair cellular signaling and disrupt normal metabolic functions. Thus, maintaining the redox balance is crucial not only for preventing oxidative/reductive damage but also for enabling proper cellular communication and function.

### 1.2. Introduction to Extracellular Vesicles

Extracellular vesicles (EVs) are small, membrane-bound particles secreted by cells into the extracellular environment, playing an increasingly recognized role in inter-organ communication and the regulation of various physiological processes [8]. EVs, which include exosomes, microvesicles, and apoptotic bodies, can carry a diverse range of biomolecules, such as proteins, lipids, nucleic acids, and organelles [9]. This cargo reflects the physiological state of the parent cell and can influence the behavior of recipient cells, making EVs critical messengers in the body’s intricate communication networks.

One of the most intriguing aspects of EVs is their ability to mediate communication between distant organs, thereby coordinating systemic responses to physiological changes. Unlike traditional signaling molecules such as hormones or cytokines, EVs provide a more complex and targeted mode of communication. They can deliver specific molecular cargo to particular recipient cells, thereby modulating cellular functions in a highly controlled manner [10]. This specificity is crucial in inter-organ communication, where precise signaling is necessary to maintain homeostasis across diverse tissues. For example, EVs released by the liver can carry metabolic information to the brain, influencing neural activity and behavior in response to changes in metabolic status [8,11]. Regarding the liver, to avoid confusion, it should be noted that liver-derived EVs and lipoproteins, although both secreted by the liver, differ significantly in their biogenesis, structure, and functions. Unlike lipoproteins, which are involved in lipid transport and feature a monolayer of phospholipids, EVs have a bilayer membrane enriched in proteins, lipids, and nucleic acids. This bilayer structure allows EVs to protect their cargo and facilitate intercellular communication [9].

Studying EVs, however, presents technical challenges due to their small size, heterogeneity, and the complexity of biological fluids. To address these challenges, various isolation and characterization techniques are recommended. For isolation, differential ultracentrifugation is widely used but may co-isolate contaminants, whereas size-exclusion chromatography (SEC) and density gradient centrifugation allow for higher purity but require careful handling. Precipitation-based kits offer fast isolation, though often at the cost of sample purity. For characterization, techniques like nanoparticle tracking analysis (NTA) and transmission electron microscopy (TEM) help determine EV size, concentration, and morphology, while Western blotting, flow cytometry, and mass spectrometry provide information on EV protein markers and molecular cargo, essential for understanding EV function and role in physiological processes [12].

In the context of redox homeostasis, EVs play a pivotal role in both propagating and mitigating oxidative stress signals across different organs. Cells under oxidative stress release EVs containing antioxidant enzymes, stress-response proteins, and signaling molecules that can influence the redox status of distant cells and tissues [13]. This allows for a coordinated response to oxidative challenges, helping to prevent localized oxidative stress from spreading and causing systemic damage. Conversely, EVs can also carry pro-oxidative signals, promoting adaptive responses in recipient cells that bolster their antioxidant defenses. This dual role underscores the importance of EVs in maintaining the delicate balance between pro-oxidant and antioxidant forces, which is essential for cellular and systemic health.

In summary, extracellular vesicles are central players in the body’s communication network, linking organs in a complex dialogue that is essential for maintaining homeostasis. Their role in regulating redox homeostasis highlights their potential as biomarkers and therapeutic tools for a variety of diseases.

This review’s purpose is to explore and summarize the emerging role of EVs as mediators of bidirectional communication between the brain and liver, particularly focusing on how redox homeostasis and its alterations can affect this communication. We chose to focus on brain-liver communication because, in the context of EVs and oxidative stress, their relationship remains unclear and underexplored. To date, no comprehensive review has examined the role of EVs in redox regulation between these two organs, highlighting the need to address this gap in the literature.

## 2. Extracellular Vesicles

### 2.1. Types of EVs, Biogenesis, and Release Mechanisms

Traditionally, they have been widely classified into two big groups—small and larEVs— just considering their size [14]. Other attempts have been made to classify vesicles based on parameters other than just their size, but this still holds many problems (due to overlapping biogenesis processes, the heterogeneous and variable content of vesicle types, and the strong influence of the microenvironment on their characteristics) [15]. One example is the effort made to find specific markers for different types of vesicles according to the organelle from which they originate. In the case of exosomes, as they come from the endocytic pathway, they are notably rich in tetraspanins like cluster of differentiation 9 (CD9), cluster of differentiation 63 (CD63), and cluster of differentiation 81 (CD81), along with elements of the endosomal sorting complex required for Transport (ESCRT), such as Tumor susceptibility gene 101 (TSG101) [16]. In contrast, the identification and characterization of markers or specific proteins for other types of EVs remain insufficiently explored. On the other hand, the redox capacity of extracellular vesicles has been studied mainly in exosomes, which show an incredible antioxidant potential due to their rich enzymatic content (e.g., superoxide dismutase, and glutathione peroxidase) [17]. Now, we will provide an overview of the main types of extracellular vesicle (EV) and summarize current knowledge about their biogenesis process [18] (Table 1).

#### 2.1.1. Small EVs

Small EVs consist of vesicles that are less than 200 nm in diameter, such as exosomes, exomeres, and a subpopulation of ectosomes/shedding microvesicles:

- EXOMERES

To start discussing small EVs, we must talk about the smallest of them all: exomeres (<50 nm). It may be problematic to classify them as EVs because they do not contain a lipid membrane or ESCRT complexes.Moreover, it is argued that they might be more closely associated with certain organelles, such as the ER, mitochondria, or the cytoskeleton [42]. They could play a role in some pathological processes like Alzheimer’s or tumor metastasis through the activity of different molecules, and they contain amyloid precursor protein (APP), β-galactoside α2,6- sialyltransferase 1 (ST6GAL1), and amphiregulin (AREG) [43]. Although some of their components are known, the mechanisms involving biogenesis remain unclear.

- EXOSOMES

Exosomes are small EVs whose size varies from 30– to 150 nm. Many cells can secrete exosomes, meaning that their content will depend on their cellular origin. Exosomes’ biogenesis starts with endocytosis and the fusion of the resulting endocytic vesicles with early endosomes. During the process of maturation from an early endosome towards a multivesicular body, invagination of the endosomal membrane occurs, giving rise to intraluminal vesicles (ILVs). These intraluminal vesicles can be driven into degradation after fusion with lysosomes or, on the contrary, be secreted into the extracellular matrix through the fusion of multivesicular bodies with the plasma membrane. After their liberation, intraluminal vesicles are termed exosomes [44,45] The maturation process of endosomes occurs thanks to the substitution in the membrane of Rab5 for Rab7, while the invagination of the membrane to form intraluminal vesicles can originate through two different processes: ESCRT-dependent or ESCRT-independent processes. ESCRTs (endosomal sorting complexes required for transport), including ESCRT-0, I, II, and III, are protein complexes that interact with each other and with other accessory proteins to form intraluminal vesicles in multivesicular bodies (MVBs) [46]. Nonetheless, there is another existing pathway to create ILVs inside MVBs, which begins with the aggregation of sphingomyelin and cholesterol into lipid rafts. Once in the rafts, sphingomyelin is hydrolyzed by neutral sphingomyelinase (nSMase), thus producing ceramides. Posterior invagination of the MVB membrane takes place due to the aggrupation of those cone-like ceramides [31]. However, MVBs formed without ESCRT machinery tend to be larger and contain fewer ILVs, often displaying irregular shapes and sizes [47]. Exosome exocytosis involves not only the cytoskeleton but also two major families of proteins: Rab GTPases, which are crucial for regulating the intracellular trafficking of MVBs [48], and Soluble NSF Attachment Protein Receptors (SNARE) proteins [49], which primarily mediate the fusion of MVBs with the plasma membrane. This fusion process requires the interaction of v-SNAREs on the MVB membrane with t-SNAREs on the plasma membrane [50].

- MICROVESICLES

Regarding microvesicles, also known as shedding ectosomes, they are a bit on the border of being considered small or large since their size can vary from 100 to 1000 nm, and their content is very numerous and widely variable, including different cellular growth factors and several enzymes such as metalloproteinases. Their biogenesis is basically due to the detachment of plasma membrane protrusions, which implies a redistribution of membrane phospholipids and the contraction of the cytoskeleton [51]. In those actions, two molecules are highly involved [33,52]: ATP binding cassette transporter 1 (ABCA1) and ADP-Ribosylation Factor 6 (ARF6) (a small GTPase) (Table 1).

#### 2.1.2. Large EVs

Large EVs are those over 200 nm in diameter, which include large oncosomes, apoptotic bodies, migrasomes, and ectosomes/shedding microvesicles.

- LARGE ONCOSOMES

Large oncosomes are a subtype of oncosomes (EVs from cancerous cells) that are characterized by being secreted from amoeboid tumor cells and having a diameter between 1000 and –10,000 nm [53]. As oncosomes, they contain an oncogenic cargo, which may help to transform the tumor microenvironment; to chang, for instance, cellular phenotype; to avoid immune response; or to transfer pro-oncogenes into recipient cells [54,55]. They can even have a role in tumor metastasis due to their content in some specific Ribonucleic Acids (RNAs), microRNAs (miRNAs), caveolin-1, metalloproteinases, and (ARF6) [53,54]. Additionally, it has also been seen that they are rich in enzymes involved in cancer-related metabolic processes, such as glyceraldehyde-3-phosphate dehydrogenase (GAPDH), lactate dehydrogenase B (LDHB), heat shock 70-kDa protein 5 (HSPA5), malate dehydrogenase (MsDH), and glucose-6-phosphate isomerase (GPI).

- APOPTOTIC BODIES

Apoptotic bodies are EVs that emerge from apoptotic cells blebbing, and their cargo specifically contains the remnants of the apoptotic process (e.g., organelles, DNA fragments, ect.). They measure from 1000 to 5000 nm. In cells undergoing apoptosis, plasma membrane blebbing is believed to occur due to caspase-3-mediated cleavage of Rho-associated coiled-coil-containing protein kinase 1 (ROCK1) [37,56]. This cleavage triggers the activation of ROCK1, which subsequently phosphorylates the myosin light chain (MLC), initiating actin–myosin contraction and causing the detachment and expansion of the plasma membrane from the cytoskeleton [57,58]. Another characteristic of these cells is their expression on the cell surface and that they are also found in apoptotic bodies of phosphatidylserine [39], which, through a series of interactions, serve as a signal for phagocytosis clearance.

- MIGRASOMES

Migrasomes are large EVs that range from 500 to 3000 nm in size, and they contain numerous small vesicles inside, resembling MVBs, except that they do not express lysosomal-associated membrane protein 1 (LAMP1), an MVB marker. However, they are abundant in N-deacetylase, N-sulfotransferase 1, and carboxypeptidase Q (all involved in migration and tumor infiltration capacity), as well as in the phosphatidylinositol glycan anchor biosynthesis class K (related to cell adhesion) and EGF domain-specific O-linked N-acetylglucosamine transferase (responsible for interaction with the extracellular matrix) [59]. All of these could potentially act as indicators for migrasomes [60]. Migrasomes have been described in several processes involving the brain and liver, for instance, ischemic brain injury or hepatocellular carcinoma [61,62].

### 2.2. Composition of EVs

#### 2.2.1. Lipid Content and Membrane Characteristics

**Figure 1:**
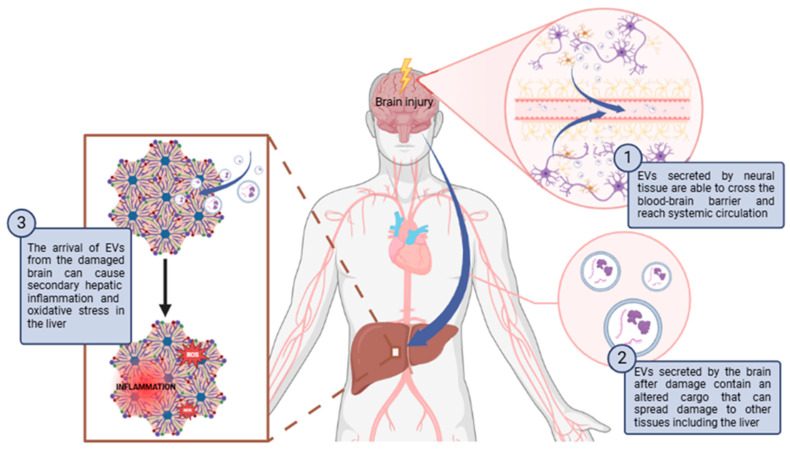
Schematic diagram of how brain damage can propagate to the liver through EVs causing secondary hepatic inflammation and oxidative stress. Abbreviations: ROS: Reactive oxygen species. Created with BioRender.com.

According to metabolomic analyses of EVs reported to date, lipids play a crucial role in the physiological functions of these vesicles and their formation [63]. Although differences have been found in the lipid composition of EV-derived vesicles, it is generally known that EVs are enriched in cholesterol, PS, sphingomyelin, and glycosphingolipids compared to the original cells hgchc [64]. Placental EVs also contain a high proportion of cholesterol and sphingomyelin. The characteristic lipid composition of the EV bilayer, as we can observe in Figure 1, contributes to their stability in different extracellular environments. Therefore, understanding this composition and the lipids that provide stability can help improve drug delivery systems [65].

Lipids, like other biomolecules, are not randomly included within EVs but are specifically classified. The membranes are enriched in sphingomyelin and cholesterol, as previously mentioned, while cholesterol and the long-saturated fatty acids of sphingolipids allow for tighter lipid packing [63]. The high cholesterol and sphingolipid content provides structural rigidity to EVs and greater resistance to physicochemical changes. It has been suggested that rigidity also depends on pH, as lower pH levels make the membrane less rigid [66]. Increased acidity makes the fluidity of EV membranes more like that of the cellular plasma membrane, which promotes fusion. It has also been suggested that several lipids are involved in the formation and release of EVs. Cholesterol plays a role in regulating the release of EVs [67], just as it is important for the release of several enveloped viruses, such as HIV-1 and the influenza virus. Similarly, ceramide is thought to be involved in the formation of ILVs within MVBs, and another group of lipids, such as lysobisphosphatidic acid (LBPA) and phosphatidic acid, is implicated in the biogenesis of EVs [64]. Beyond their essential structural role in membranes, lipids may also contribute to reproduction, as seminal EVs are thought to interact with sperm cells, transferring lipids like cholesterol, which are vital for the capacitation process [68]. Given that lipids serve as fundamental structural and functional components of EVs, further lipidomic studies on EVs from various cell types and bodily fluids are necessary to clarify the role of lipids in EV biogenesis and their biological functions.

#### 2.2.2. Nucleic Acids

The presence of functional RNA in EVs was first described in 2006 for EVs derived from murine stem cells and in 2007 for those derived from murine mast cells taken up by human mast cells [69,70]. It was found that the RNA patterns in exosomes were significantly different from those in their parent cells. A large amount of mRNA and miRNA were notably enriched or even exclusively present in the exosomes, indicating the existence of a specific mechanism for selecting and targeting these RNAs to the vesicles. While cellular mRNA varies in size between 400 and 12,000 nucleotides (nt), RNA in EVs does not exceed 700 nt [71]. This could be due to the purity of the sample studied, as the amount of RNA in EVs varies depending on the type of originating cell. Various studies show that exosomes are composed of messenger RNA (mRNA) and microRNA (miRNA), which can be unidirectional and transferred between cells [69]. They can also contain long non-coding RNA, piwi-interacting RNA [72], ribosomal RNA (rRNA), and tRNA fragments [73]. To elaborate, miRNAs are a type of non-coding RNA (ncRNA) about ∼22 nucleotides (nt) in length that functionally repress target mRNA by binding to their 3′ untranslated regions [74]. They are involved in various biological processes such as differentiation, apoptosis, proliferation, and development [75]. Interestingly, the mRNA content of EVs is modulated by stress conditions and the physiological state of the cell, potentially playing a key role in maintaining tissue homeostasis, synchronizing the functional state of cells, and facilitating tissue repair [76].

The DNA in extracellular vesicles is known as EV-DNA. Its size can vary between 100 base pairs and 2.5 kilobase pairs, which are found within EVs [77]. Mitochondrial DNA (mtDNA), single-stranded DNA, double-stranded DNA (dsDNA), and oncogenic amplifications (such as c-Myc) have been detected in EVs. It has been shown that depending on the EV subgroup, they can carry different DNA loads [78]. Through EVs, mtDNA migration can occur, meaning that these EVs may represent an alternative pathway through which altered mtDNA can enter other cells and promote the spread of various pathologies [79]. The fact that the DNA transported by EVs can be used to identify mutations in parental tumor cells illustrates its significant potential as a translational biomarker. However, the physiological significance of DNA loading in EVs is currently unknown.

#### 2.2.3. Proteins

The protein composition of EVs can be influenced by the type of parent cell and the mechanism of biogenesis [80,81,82]. However, numerous proteins, such as MHC II, ESCRT proteins, heat shock chaperones, and Alix, are found in EVs regardless of their cell of origin and are commonly used as general EV markers [80]. Research indicates that exosomes, compared to their parent cells, are enriched in transmembrane proteins and glycoproteins [81]. The protein composition of EVs is heterogeneous and depends on various factors, such as physiological conditions or cell type, among others. This diversity allows EVs to perform specific cellular communication functions and regulate biological processes [83]. Some EV proteins can also be organized into functional categories. Mass spectrometry analysis of EVs derived from ovarian cancer cell lines has shown an enrichment of proteins that undergo modifications like phosphorylation and acetylation. Among the most enriched were proteins such as mitogen-activated protein kinase (MAPK), phosphatidylinositol-3-kinase, and members of the ErbB family [81]. These proteins are central players in cellular signaling pathways that regulate critical processes like cell growth, survival, and differentiation. The presence of these proteins suggests that EVs might modulate fundamental processes in tumor progression [84,85,86]. Additionally, the surface proteins on EVs can reflect the biological state of their parent cells. EV-associated proteins, including receptors, transcription factors, and enzymes, may retain their functionality and induce phenotypic changes in recipient cells.

#### 2.2.4. Metabolites

EVs encapsulate a diverse range of metabolites, which play significant roles in physiological and pathological processes. These metabolites include amino acids, lipids, sugars, nucleotides, and organic acids, among others, which reflect the metabolic state of the parent cells and provide valuable biomarkers for disease diagnosis and monitoring [87,88] Metabolites within EVs contribute to various cellular functions, such as energy production, signaling, and macromolecule synthesis. For example, exosomal metabolites such as glutamate and lactate have been shown to support cancer cell survival in hypoxic conditions by modulating metabolic pathways [87]. Regarding oxidative stress, Vs have been found to contain antioxidant metabolites such as coenzyme Q10, which not only mitigates oxidative damage but also modulates gene expression related to redox signaling [88]. Conversely, EVs derived from stressed or damaged cells may carry molecules that exacerbate oxidative stress in recipient cells, contributing to pathological conditions such as neurodegeneration or cancer progression. The role of EV metabolites extends to immune modulation and the regulation of inflammatory responses. Specific metabolic profiles of EVs, such as those enriched in polyunsaturated fatty acids or adenosine, have been linked to the immune evasion strategies of cancer cells and other pathological states [87]. Recent advances in metabolomics, using techniques like mass spectrometry and NMR spectroscopy, have enhanced our ability to characterize the metabolite profiles of EVs. These profiles vary according to cell type, environmental conditions, and disease state, providing insight into cellular metabolism and potential intercellular communication pathways [88].

#### 2.2.5. Organelles

Extracellular vesicles (EVs) are highly heterogeneous and contain diverse molecular and structural components, including the presence of organelle-related materials. Among these, apoptotic bodies—one of the EV subtypes—stand out for their ability to encapsulate intact organelles such as fragments of nuclei, mitochondria, and even other cytoplasmic organelles during the process of programmed cell death. These apoptotic bodies range in size from 50 to 5000 nm and carry not only organelle-derived materials but also other biomolecules that can influence intercellular communication [89,90]. The presence of organelle-derived components in EVs is not limited to apoptotic bodies. Studies have also identified mitochondrial proteins and DNA within exosomes and microvesicles, highlighting their role in cellular signaling and stress responses. Such mitochondrial components, particularly when released under stress conditions, can modulate immune responses or contribute to pathological states, such as inflammation or cancer progression. These findings underline the potential of EVs to act as carriers of organelle-derived cargos, which may serve as biomarkers or therapeutic targets in diseases associated with organelle dysfunction [89].

### 2.3. Functions in Intercellular Communication

Throughout evolution, both prokaryotes and eukaryotes have developed sophisticated strategies for intercellular communication. EVs have been recognized as important vehicles for intercellular communication due to their ability to transfer proteins, nucleic acids, and lipids, influencing various physiological and pathological functions in both recipient and donor cells [73]. While research has focused more on the role of EVs in pathological processes such as cancer and autoimmune diseases, other aspects, such as the maintenance of homeostasis mediated by EVs or the regulation of physiological functions, require further study. The number of EVs released and uptaken likely depends on a variety of factors, such as microenvironmental conditions, the type of donor and recipient cells, and their physiological state [91,92,93].

The ability to bind or fuse with the target cell membrane allows exosomes to deliver exosomal surface proteins and possibly cytoplasm to the recipient cell [94,95]. Therefore, EVs carry biologically active molecules as cargo from their producing cell.

Thanks to their ability to horizontally transfer these macromolecules, EVs have become important mediators of intercellular communication and are valuable in therapeutic studies. Using EVs as nanocarriers opens the possibility of specific bioengineering to transport therapeutic cargo molecules [73].

## 3. Brain–Liver Axis

### 3.1. Physiological Connections Between the Brain and Liver

The physiological connections between the brain and liver are recognized as a complex and dynamic interplay, often referred to as the liver–brain axis. This axis is mediated by different elements that participate in the communication between these two organs, and it occurs via the signaling between the nervous and circulatory systems, including neural pathways and endocrine signaling, respectively [96,97].

The vagus nerve is a primary conduit for communication between the brain and liver, containing both afferent and efferent fibers that facilitate bidirectional signaling. Afferent fibers transmit sensory information from the liver to the brain, providing feedback on metabolic states, while efferent fibers convey signals from the brain to the liver, influencing hepatic functions such as glucose metabolism and lipid synthesis [96,97]. Neurotransmitters from the brain, such as serotonin, dopamine, and norepinephrine, also play crucial roles in this brain–liver axis. For instance, serotonin has been implicated in the regulation of liver metabolism, influencing the secretion of bile and the activity of cytochrome P450 enzymes, which are essential for drug metabolism and the processing of endogenous compounds. Additionally, dopamine and norepinephrine are involved in modulating hepatic glucose production and lipid metabolism [98]. Moreover, the liver’s ability to produce and release various metabolites, such as glucose and ketones, is influenced by the brain’s signaling. This interaction is particularly evident in the context of dietary changes, where alterations in nutrient composition can affect neurotransmitter levels and, consequently, liver metabolism [99].

On the other hand, there are several molecules secreted by the liver that modulate brain function. For instance, Fibroblast Growth Factor 21 (FGF21); is a liver-derived hormone that plays a pivotal role in the context of metabolic regulation. It is secreted in response to glucose metabolic disorders and has been shown to influence brain energy balance and neuronal function. Also, FGF21 modulates the astrocyte-neuron lactate shuttle, enhancing neuronal protection and metabolic support during periods of energy stress [100]. Another molecule secreted by the liver that modules brain function is Leptin. This hormone is primarily produced by adipocytes but also secreted by the liver, playing a significant role in regulating energy balance and appetite. It acts on the hypothalamus to suppress appetite and increase energy expenditure [101]. Moreover, bile acids, produced in the liver, have emerged as important signaling molecules that influence brain function. They can cross the blood–brain barrier and interact with central nervous system receptors, modulating neuronal activity and potentially influencing mood and cognition. Bile acids have been shown to activate the hypothalamic–pituitary–adrenal (HPA) axis, affecting stress responses and metabolic regulation [102].

### 3.2. Role of EVs in Mediating This Communication

EVs have emerged as critical mediators of intercellular communication, playing a significant role in the relationship between the liver and the brain. This is possible because EVs can cross the blood-brain barrier (BBB) [103,104]. EVs from the periphery enter the brain through receptor-mediated transcytosis, involving pathways such as dynamin-, clathrin-, and caveolin-dependent endocytosis, with specific receptors like transferrin and C-type lectins playing a key role. This process is enhanced during inflammation or increased neuronal activity. Conversely, brain-derived EVs cross into the bloodstream, particularly during pathological states like neurodegeneration or injury, where barrier integrity is compromised [104,105]. EVs released by brain cells, such as neurons and glial cells, can cross the BBB into the bloodstream primarily through receptor-mediated transcytosis in brain endothelial cells. However, in the absence of inflammation or barrier disruption, this exchange is minimal, likely due to the stringent physical and functional barriers imposed by the BBB to preserve brain homeostasis. Consequently, the release of brain-derived EVs into systemic circulation is more frequently observed in pathological states or when BBB integrity is compromised [105]. This section explores physiological and pathophysiological processes by which EVs mediate communication between the liver and the brain.

EVs participate in physiological communication between the liver and the brain in the transport of the Cytochrome P450 (CYP) enzyme family. Cytochrome P450 (CYP) enzymes are crucial in the liver’s ability to detoxify a wide range of substances, including drugs, toxins, and metabolic byproducts. Traditionally, CYP enzymes have been studied in the liver, where they perform most of the body’s detoxification functions. However, recent research suggests that the activity of CYP enzymes is not restricted to the liver. Studies have shown that EVs, originating from the liver, can carry various bioactive molecules, including CYP enzymes, to distant organs, including the brain. This communication between the liver and brain through EVs allows the presence of CYP enzymes in the brain, which could provide a neuroprotective mechanism by detoxifying potentially harmful compounds that reach the central nervous system [106,107,108]. Although liver-derived EVs have been shown to cross into the brain under normal physiological conditions, the transport of brain-derived EVs to the liver appears to be restricted by the stringent regulation of the BBB and is primarily associated with pathological disruptions.

Aside from these physiological processes, there is evidence that EVs mediate the crosstalk between the liver and the brain in some pathological processes [106]. The studies that relate communication between the brain and liver via EVs will be developed in Section 4.2 and Section 4.3. of this review, but in general, brain-derived EVs can induce oxidative stress and inflammation in the liver, especially following traumatic brain injury (TBI) or brain inflammation. These EVs cross the blood-brain barrier, activating inflammatory pathways and promoting oxidative damage in liver tissues. Conversely, liver-derived EVs also affect the brain, as seen in hepatic ischemia-reperfusion injury and hepatic encephalopathy, where they induce neuroinflammation and oxidative stress, contributing to neurodegenerative diseases like Alzheimer’s. Both brain-liver and liver-brain EV exchanges create a harmful cycle of oxidative stress in both organs.

## 4. Redox Homeostasis and Brain–Liver Communication via EVs

### 4.1. Influence of Oxidative Stress on EVs Biogenesis and Composition

The biogenesis and composition of EVs are influenced by the redox homeostasis of the cells from which they originate. Excess ROS can disrupt cellular signaling, affecting the biogenesis and molecular composition of EVs. These EVs can carry oxidized lipids and proteins, potentially causing harmful effects in target cells. However, EVs generated under oxidative stress also contain antioxidant molecules that help regulate the oxidative response in recipient cells, protecting them from further damage [109,110,111]. This section explores how oxidative stress alters the biogenesis and composition of EVs released from brain and liver cells, highlighting the implications for cellular communication and physiological processes.

#### 4.1.1. Brain-Derived EVs

Firstly, it is necessary to explore how the oxidative stress of brain cells affects the biogenesis and release of EVs. Exosomes are secreted by several brain cells, including neurons, astrocytes, and microglia, and it has been reported that their release is increased by oxidative stress [112,113]. However, studies such as that of Zhu L et al. [114] claim that the induction of oxidative stress in an in vitro model of human astrocytes reduces exosome release but increases their size. In other brain cells, such as brain microvascular endothelial cells, Vujić T et al. [115] observed that the induction of oxidative stress with morphine in an in vitro experiment does not change the size of EVs. On the other hand, there are studies observing changes in the biogenesis of brain cells, unlike in other cell types where oxidative stress was found to cause changes in membrane lipids and proteins [111]. It seems that there is no consensus on how changes in the redox imbalance affect the biogenesis and release of EVs from brain cells, as this depends on the cell type and the origin of the damage.

Moreover, there are several studies in which it has been observed how oxidative stress changes EV cargo from brain cells. EVs released under oxidative stress can mediate either protective or harmful signals in target cells, depending on their specific biochemical cargo. In the context of neurodegenerative diseases, oxidative stress plays a critical role in the pathogenesis of these diseases. For instance, Alzheimer’s disease (AD) is characterized by persistent neuronal inflammation and oxidative stress, both of which are closely linked to the formation of amyloid-beta (Aβ) plaques and neurofibrillary tangles. These changes progressively impair neuron function, ultimately leading to cell death. It has been suggested that oxidative stress not only contributes to Aβ production and accumulation but is also exacerbated by it, creating a reinforcing cycle [111]. Aβ that accumulates can be expelled from cells through exosomes, which promotes Aβ aggregation and triggers neuroinflammation and oxidative stress in surrounding neurons [116]. You et al. [117] found that astrocytes from postmortem human brains of AD patients release EVs that contain specific disease-related proteomes, which can cause the spread of the disease to other cells. Oxidative stress can also change the miRNA content of EVs. For instance, McKeever PM et al. [118] have observed differences in the levels of miRNAs of exosomes derived from cerebrospinal fluid (CSF) of young-onset AD patients compared to healthy subjects, such as an upregulation of miR-125b-5p. It has been reported that miR-125b-5p leads to a significant overexpression and hyperphosphorylation of tau, indicating a connection between this miRNA and the progression of AD [119]. However, there are other studies in which a protective effect of EVs released under oxidative stress has been observed. Wang S. et al. [120] and Fröhlich D. et al. [121] have shown that the induction of oxidative stress in a primary culture of astrocytes and oligodendrocytes causes a change in the charge of EVs of these cells, such as an increase in synapsin 1 or antioxidant enzymes. EVs from these cells reach their target cells, i.e., neurons, and induce protective effects, promoting neuronal resistance to oxidative stress and neuronal survival and growth.

#### 4.1.2. Liver-Derived EVs

The biogenesis and composition of liver-derived EVs can be influenced by oxidative stress. In the case of biogenesis and release of EVs, two studies by Van Meteren N et al. [122,123] have demonstrated that oxidative stress induced by polycyclic aromatic hydrocarbons (PAH) increases the release and alters the biogenesis of EVs in hepatocytes. PAH treatment leads to a reduction in total cellular cholesterol content and an increase in membrane fluidity, which may facilitate EV release. Moreover, EVs released from PAH-treated hepatocytes contain higher levels of cholesterol and ESCRT machinery proteins compared to those from untreated cells. Oxidative stress not only increases the release of EVs but also alters their cargo, which can stimulate immune cells [110]. One of the key components that hepatocyte-derived EVs carry under oxidative stress is mtDNA. In an alcoholic hepatitis mice model, where oxidative stress exists due to the generation of ROS in the liver [124], EVs containing mtDNA released by hepatocytes have been shown to activate Toll-like receptor 9 (TLR9), which is highly expressed in immune cells. The activation of TLR9 by mtDNA triggers a pro-inflammatory response, leading to neutrophilic inflammation, which exacerbates liver injury and promotes the progression of alcoholic hepatitis [125]. Another example of the change in EV cargo under oxidative stress in alcoholic hepatitis is the increase in miR-122. Exosomes containing miR-122 from hepatocytes can inhibit the expression of heme oxygenase-1 in monocytes. This enzyme has a protective enzyme with anti-inflammatory properties. As a result, monocytes become more sensitive to lipopolysaccharides (LPSs) and produce pro-inflammatory cytokines such as TNFα and IL-1β, further driving liver inflammation. [126]. Also, in an in vitro model, mouse and human hepatocytes treated with palmitate to induce lipotoxicity and oxidative stress release EVs that express TNF-related apoptosis-inducing ligand (TRAIL) on their surface, which were recognized by death receptor 5 on macrophages, resulting in IL-6 release which contributes to liver inflammation and immune cell activation [127]. EVs derived under oxidative stress from hepatocytes can not only increase hepatitis by activating cells of the immune system but also increase liver fibrosis by activating hepatic stellate cells (HSCs). Povero D. et al. [128] have observed how the EVs of the hepatocytes of high-fat diet-treated mice, a model of NASH in which oxidative stress occurs, contain miR-128-3p that activates HSCs. Table 2 summarizes all these results. 

### 4.2. Role of Brain-Derived EVs in Modulating Liver Redox Homeostasis and Its Pathophysiological Implications

Brain-derived EVs have been shown to affect the liver in pathological situations. Brain damage can propagate to the liver through EVs (Figure 1). For instance, Hazelton I. et al. [129] observed in a mouse model of TBI that neuronal injury activated microglia to release EVs. These EVs were able to cross the damaged BBB and increased in plasma. The exogenous administration of EVs isolated from TBI mouse plasma caused secondary hepatic inflammation. In another study, the injection of EVs derived from the brains of TBI mice into healthy mice induced inflammation in other tissues, including the liver, along with tissue damage and increased hepatocyte senescence. [130]. On the other hand, in another work, it was observed that cerebral inflammation induced by IL-1β increased the secretion of astrocyte-derived EVs, which traveled to the liver and caused hepatic inflammation by increasing the expression of IL-1β and TNF-α, and the infiltration and activation of neutrophils [131]. Lin H. et al. [132] also observed that EVs derived from the brains of rats with sepsis produced damage and inflammation in the liver of healthy rats. Secondary hepatic inflammation derived from the arrival of EVs from the damaged brain can cause oxidative stress in the liver. The infiltration and activation of inflammatory cells, such as neutrophils in the tissues, leads to the release of proinflammatory cytokines and other inflammatory mediators that induce cell damage and increase the production of ROS and RNS. This results a the loss of redox balance in the liver and the development of oxidative stress. At the same time, oxidative stress increases inflammation, causing a vicious circle in which it promotes the pathogenesis of liver diseases [133,134].

In general, it has been mainly studied how damage in the brain changes the composition of EVs, propagating damage to other organs, such as the liver, and causing inflammation, possibly leading to the appearance of oxidative stress. However, there are no studies in which a protective effect of EVs from the brain on the liver is observed. It has indeed been observed that EVs derived from neural progenitor cells can reach the liver in a murine model [135]. Although there is no evidence that these EVs can affect the redox homeostasis of the liver, they have certainly been shown to exert protective effects against oxidative stress in the brain itself [136,137] and in other tissues [138]. It is, therefore, possible that this also occurs in the liver. Given that EVs from neural progenitor cells have shown protective effects against oxidative stress in the brain and other tissues, future research should investigate their potential to counteract liver damage, offering promising therapeutic avenues for mitigating liver diseases linked to brain injury.

### 4.3. Role of Liver-Derived EVs in Modulating Brain Redox Homeostasis and Its Pathophysiological Implications

**Figure 2:**
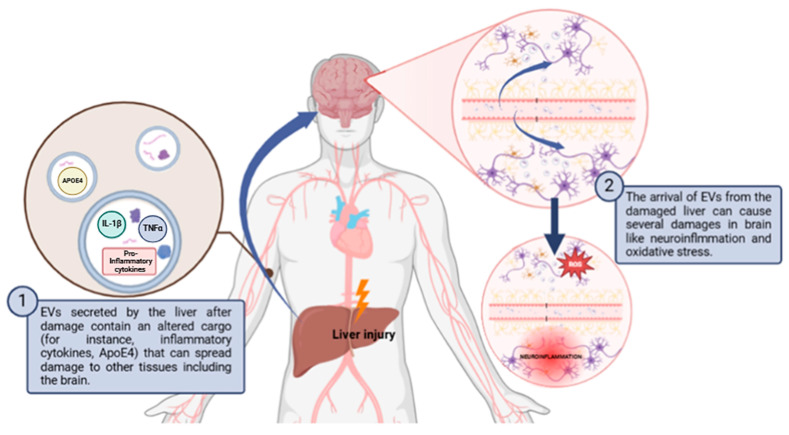
Schematic diagram of how liver damage can propagate to the brain through EVs causing neuroinflammation and oxidative stress. Abbreviations: ApoE4: apolipoprotein E4; ROS: reactive oxygen species. Created with BioRender.com.

As discussed in the previous section, it has been observed that liver-derived EVs are also capable of altering the redox homeostasis of the brain in a pathological context (Figure 2 and Table 3). Exosomes isolated from the serum of rats with hepatic ischemia–reperfusion injury (HIRI) were shown to induce neuronal damage in the hippocampus and cortex in healthy rats. The exosomes were transported to the hippocampus and cortex and caused the activation of NLRP3 inflammasome and caspase-1 pathway, promoting oxidative stress and initiating neuronal pyroptosis [139]. Moreover, hepatic encephalopathy demonstrates the propagation of oxidative stress from the liver to the brain via EVs. This disease is characterized by a significant neurological impairment in the terminal stages of severe liver disease. Hyperammonemia is the main contributor to neurological dysfunction in cirrhotic patients [106,140]. When EVs from hyperammonemic rats were injected into normal rats, these EVs reached the cerebellum and induced neuroinflammation and motor incoordination by altering Purkinje neurons and microglia. The EVs produced activation of microglia via the TNFR1/NF-κB/GABA transporter 3 (GAT3) pathway, leading to the accumulation of proinflammatory factors like IL-1β, TNF-α, and CD68 [141,142]. Similar to what occurs in the liver, neuroinflammation resulting from the accumulation of these proinflammatory factors leads to the generation of ROS and thus to the occurrence of oxidative stress in the brain, which is related to the pathogenesis of neurodegenerative diseases [143] On the other hand, age-related thyroid deficiency has also been shown to increase exosomal transport of ApoE4 from the liver to the brain in a murine model [144]. This can lead to neuroinflammation, beta-amyloid aggregation, and mitochondrial dysfunction in ApoE4 allele carriers, resulting in increased ROS and oxidative stress. This finally results in AD-related dementia [141].

Physiologically, excluding the pathological context, there are mechanisms by which liver-derived EVs can affect brain oxidative stress. Royo et al. [145] demonstrated that isolated EVs secreted by hepatocytes can modify, in a murine model, the blood metabolome and endothelial function, suggesting that these vesicles play a role in systemic redox balance. The arginase-dependent mechanism identified in their study indicates that liver-derived EVs may influence nitric oxide production, which is crucial for maintaining vascular homeostasis and modulating oxidative stress levels in the brain.

In summary, we propose that liver-derived EVs play a significant role in altering the redox homeostasis of the brain in both pathological and physiological contexts. Their ability to induce neuroinflammation and oxidative stress in conditions such as hepatic encephalopathy and age-related thyroid deficiency highlights their impact on brain health. Therefore, understanding the mechanisms of action of these EVs could offer new opportunities to address neurodegenerative diseases and related disorders.

## 5. Conclusions and Future Directions

EVs have emerged as pivotal mediators in the complex communication between the liver and brain. This review proposes a bidirectional role of the liver- and brain-derived EVs in influencing redox homeostasis in both physiological and pathological contexts, either promoting oxidative stress and inflammation or, potentially, in offering protection against such damage. In pathological conditions like TBI, hepatic encephalopathy, and neurodegenerative diseases, EVs can potentially propagate inflammation and oxidative stress between the liver and brain, exacerbating damage and creating a feedback loop of redox homeostasis imbalance.

Despite the harmful impact of EV-mediated crosstalk on disease, the potential for EVs to serve as therapeutic agents is growing [146] Future research should explore the protective capabilities of specific EV populations, particularly those derived from healthy or younger cells, to counteract liver–brain damage. Additionally, understanding the specific molecular cargo of EVs, which varies based on the redox state and origin of the cells, is crucial for developing targeted therapies.

The therapeutic use of EVs offers a promising approach to managing diseases associated with oxidative stress and inflammation in the liver and brain. Moving forward, a deeper understanding of the mechanisms by which EVs influence redox homeostasis is needed, particularly regarding their dual role in promoting or mitigating damage. Therapeutic strategies could involve either inhibiting pro-oxidative EVs or harnessing the regenerative potential of EVs with protective properties. Furthermore, exploring EVs as drug delivery vehicles or isolating beneficial components from EVs, such as specific proteins, miRNAs, or lipids, could accelerate their application.

## Figures and Tables

**Table 1:** Molecules involved in the biogenesis of different types of EVs.

Type of EVs	Regulatory Molecule	References
1. Exosomes	Rab5/VPS21; VPS-HOPS; Rab7; HRS, STAM; TSG101; VPS 4, 22, 25, 28, 36, 37; UBAP1; CHMP 2,3,4,5,6; IST1; ALIX; VTA1; Syntenin; Syndecan; Sphingomyelin; nSMase and Ceramide	[19,20,21,22,23,24,25,26,27,28,29,30,31]
2. Microvesicles	Actin-myosin; ABCA1; ARF6; PLD; ERK; MLCK; ARRDC1; CD81; TSG101; CHMP 2,3,4,5,6; IST1 and ALIX	[22,24,27,32,33,34]
3. Large oncosomes	Actin-myosin, MRCK, and ROCK	[35,36]
4. Apoptotic bodies	Actin-myosin, ROCK1, TMEM16F, Xkr8, and Caspase-3	[33,37,38,39,40]
5. Migrasomes	TSPAN4	[41]

Abbreviations: MRCK: myotonic dystrophy kinase-related cdc42-binding kinase; ROCK1: Rho-associated protein kinase 1; TMEM16F: transmembrane protein 16F; Xkr8: XK-related 8; Caspase-3: Cysteine-aspartic acid protease 3; TSPAN4: tetraspanin 4; ABCA1: ATP-binding cassette subfamily A member 1; ARF6: ADP-ribosylation factor 6; PLD: phospholipase D; ERK: extracellular signal-regulated kinase; MLCK: myosin light-chain kinase; ARRDC1: arrestin domain-containing protein 1; CD81: cluster of differentiation 81; TSG101: tumor susceptibility gene 101; CHMP 2,3,4,5,6: charged multivesicular body protein 2, 3, 4, 5, 6; IST1: increased sodium tolerance 1; ALIX: ALG-2 interacting protein X; Rab5/VPS21: Ras-related protein Rab-5, vacuolar protein sorting-associated protein 21; VPS-HOPS: vacuolar protein sorting-homotypic fusion and protein sorting complex; Rab7: Ras-related protein Rab-7; HRS: hepatocyte growth factor-regulated tyrosine kinase substrate; STAM: signal transducing adaptor molecule; vPS 4: Vacuolar protein sorting 4; UBAP1: ubiquitin-associated protein 1; VTA1: vacuolar protein sorting-associated protein 1; nSMase: neutral sphingomyelinase.

**Table 2:** Influence of oxidative stress on brain-derived EVs and liver-derived EVs biogenesis and composition. Abbreviations: Ref: Reference; Nrf2: Nuclear factor (erythroid-derived 2)-like 2; AD: Alzheimer’s Disease; CSF: Cerebrospinal Fluid of Patients; KCl: Potassium chloride; ESCRT: Endosomal sorting complexes required for transport; TLR9: Toll-like receptor 9; DR5: Death receptor 5; TRAIL: TNF-related apoptosis-inducing ligand; NASH: Non-alcoholic steatohepatitis.

Model and Cell/Tissue Type of Origin	Type of EVs	Condition	Oxidative Stress Markers	EV Biogenesis or Release Change	Cargo	Effect	Ref.
**Brain-Derived EVs**							
An in vitro model human retinal astrocytes	Exosomes	Tert-butyl hydroperoxide (tBHP)	Dichloro-dihydro-fluorescein diacetate	EVs increase in size and reduce their release	Unknown	Unknown	[114]
An in vitro model of Human Brain Microvascular Endothelial Cells	Exosomes	Morphine	Proteins involved in the Nrf2 pathway (previous study)	EV size does not change	Unknown	Unknown	[115]
Postmortem human brains from AD patients	Exosomes	AD	-	-	Increased levels of amyloid-beta oligomers	Neuronal toxicity in human neuroblastoma SH-SY5Y cell line	[116]
An in vitro model of Astrocytes from AD patients	Small-EVs	AD	-	-	Disease-related proteome. Elevated integrin-β1	Unknown	[117]
CSF from AD patients	Exosomes	AD	-	-	miR-125b-5p	Unknown	[118]
An in vitro model of Astrocytes from the cerebral cortex of newborn C57BL/6 mice	Exosomes	-High KCl concentration -Hydrogen peroxide	Demonstrated in previous literature	-	Synapsin 1	Promotes neurite outgrowth and neuronal survival	[120]
An in vitro model of Oligodendrocyte from C57Bl/6-N embryonic mice	Exosomes	Hydrogen peroxide	Demonstrated in previous literature	-	Superoxide dismutase and catalase	Promote neuronal survival and help cells to resist induced oxidative stress	[121]
**Liver-Derived EVs**							
An in vitro model of: -Primary rat hepatocytes -Human/rat hepatocytes of the WIF-B9 cell line	Unknown	Polycyclic aromatic hydrocarbons (PAH)	Dihydroethidium (DHE)	-Biogenesis with higher levels of cholesterol and ESCRT proteins -EVs increase their release	Unknown	Unknown	[122,123]
Liver from a mice model	Unknown	Chronic-plus-binge alcohol drinking	Demonstrated in previous literature	-	mtDNA	TLR9-mediated neutrophilic inflammation	[125]
Liver from a mice model	Exosomes	Alcoholic hepatitis	Demonstrated in previous literature	-	miR-122	Sensitize monocytes to LPS	[126]
An in vitro model of: -Primary mouse hepatocytes -Primary human hepatocytes -Huh7 hepatocytes	Small-EVs	Saturated fatty acid-induced lipotoxicity	Demonstrated in previous literature	EVs increase their release	TRAIL	DR5-dependent macrophage activation	[127]
Primary hepatocytes from a mice model	Unknown	High- fat diet-treated mice (NASH model)	Demonstrated in previous literature		miR-128-3p	Activation HSCs	[128]

**Table 3:** Redox homeostasis and brain–liver communication via EVs. Abbreviations: TBI: traumatic brain injury; HSP70: heat shock protein 70; HIRI: hepatic ischemia–reperfusion injury; ApoE4: apolipoprotein E4.

Origin of EVs	Type of EVs	Disease or Disease Model	EVs Cargo	Destination	Effects Related to Redox State Change	Ref.
**Brain-Derived EVs**						
Mouse brain microglia	Unknown	TBI	Unknown	Liver	Macrophages and neutrophils are recruited to induce hepatic inflammation.	[129]
Mouse brain	Unknown	TBI	Unknown	Liver	Hepatic inflammation, tissue damage and hepatocyte senescence.	[130]
Mouse brain astrocytes	Exosomes	IL-1β induced inflammation	Different miRNAs and proteins that have PPARa as a target.	Liver	IL-1β ↑, TNF-α ↑, hepatic inflammation.	[131]
Rat brain	Unknown	Sepsis	Unknown	Liver	Damage and inflammation	[132]
Primary mouse neural progenitor cells	Exosomes	None	HSP70 protein and miR-20a, miR-26b, miR-124 miRNAs	Systemic	Unknown effects on the liver; protective effect on the brain	[135]
**Liver-Derived EVs**						
Rat liver	Exosomes	HIRI	Unknown	Brain (hippocampus and cortex)	Oxidative stress (ROS increase) and neuronal pyroptosis	[139]
Rat liver	Unknown	Hyperammonemic rats	IL-1β, TNFα and other inflammatory factors	Bran and cerebellum	Activation of microglia leading to neuroinflammation	[141,142]
Liver	Exosomes	Age-related thyroid deficiency	ApoE4	Brain	Neuroinflammation, beta-amyloid aggregation and mitochondrial dysfunction	[144]
Primary rat hepatocytes	Small-EVs	None	Arginase	Systemic	Influence nitric oxide production, maintaining vascular homeostasis and modulating oxidative stress levels	[145]
